# Immunoglobulin A vasculitis: The clinical features and pathophysiology

**DOI:** 10.1002/kjm2.12852

**Published:** 2024-06-03

**Authors:** Ya‐Chiao Hu, Yao‐Hsu Yang, Bor‐Luen Chiang

**Affiliations:** ^1^ Department of Pediatrics National Taiwan University Hospital Taipei Taiwan; ^2^ Graduate Institute of Clinical Medicine, College of Medicine, National Taiwan University Taipei Taiwan; ^3^ Genome and Systems Biology Degree Program College of Life Science, National Taiwan University Taipei Taiwan; ^4^ Medical Research National Taiwan University Hospital Taipei Taiwan

**Keywords:** aberrant‐glycosylated IgA, anti‐endothelial cell antibody, COVID‐19, immunoglobulin A vasculitis, pathophysiology

## Abstract

Palpable purpura, gastrointestinal symptoms, joint involvement, and renal disease characterize immunoglobulin A vasculitis (IgAV). Renal involvement ranging from mild proteinuria to severe nephritic or nephrotic syndrome highlights the importance of monitoring kidney function in patients with IgAV. Recognizing these key features is crucial for early diagnosis and appropriate management to prevent long‐term complications related to kidney disease. However, the pathogenesis of IgAV remains unclear. Disease mechanisms involve various factors, including the interplay of aberrantly glycosylated IgA, anti‐endothelial cell antibodies, and neutrophils following infection triggers, which are the main pathogenic mechanisms of IgAV. Insights from cases of IgAV related to Coronavirus disease 2019 have offered additional understanding of the connection between infection and IgAV pathogenesis. This review provides a valuable resource for healthcare professionals and rheumatology researchers seeking a better understanding of the clinical features and pathophysiology of IgAV.

## 1 INTRODUCTION

Immunoglobulin A vasculitis (IgAV), previously known as Henoch‐Schönlein purpura, is a leukocytoclastic vasculitis that predominantly affects small vessels. In children and adolescents, the disease is one of the most common types of systemic vasculitis, characterized by typical purpura with potential involvement of the gastrointestinal (GI) tract, joints, or kidneys.^1^ Some rheumatologists may overlook the disease as most patients have a self‐limiting course or achieve remission within a month after treatment. Nevertheless, a small proportion of patients with IgAV nephritis (IgAVN) have a refractory course and develop chronic kidney failure or end‐stage renal disease.^2^, ^3^, ^4^

Although the epidemiology, clinical manifestations, and outcomes of IgAV are well‐established, the pathophysiology of the disease remains unclear. Over the past decade, efforts have been made to understand IgAV pathogenesis. Discoveries related to environmental and genetic elements and the acknowledgment of aberrant IgA can offer valuable insights into this matter.^5^, ^6^ However, this mechanism does not apply to all patients with IgAV. During the Coronavirus disease 2019 (COVID‐19) pandemic, several studies have demonstrated the pathophysiological findings of COVID‐19‐related IgAV,^7^ contributing to a broader understanding of the disease.

In this article, we provide some updates on epidemiology, clinical features, and diagnosis of IgAV. We also highlight recent advances in the pathophysiology of IgAV and share experiences related to COVID‐19‐associated IgAV.

## 2 UPDATE OF THE CLINICAL FEATURES OF IgAV

### 2.1 Epidemiology of IgAV

Globally, approximately 90% of the patients with IgAV infection are children. The annual incidence rate of IgAV in children and adolescents under 17 years of age ranges from 6.79 to 55.9 per 100,000 children across different countries,^8^, ^9^, ^10^, ^11^, ^12^, ^13^ whereas a few population studies report an incidence rate of 0.1–0.8 per 100,000 in adults. According to our preliminary analysis of the Taiwan National Health Insurance Research Database (NHIRD) from 2012 to 2019, the incidence of IgAV among children younger than 17 years old was 8.3 per 100,000 people per year. The wide variation in incidence rates may be attributed to the use of various data sources across different regions and variations in ethnicities. The true incidence of IgAV might be underestimated in epidemiological results from healthcare databases, as some pediatric patients with isolated skin presentations do not require hospitalization or medication. Similar to other diseases, a change in the incidence of IgAV has been observed during the COVID‐19 period. Limited studies from Korea, Japan, and Turkey report a decrease in newly diagnosed cases of IgAV during the pandemic,^8^, ^9^, ^10^ potentially due to a decline in other viral infections.

### 2.2 Clinical presentations and diagnostic strategy in IgAV

Unlike other types of systemic vasculitis, IgAV in children commonly follows a self‐limiting course.^1^ Typical palpable purpura over the dependent part of the body, without thrombocytopenia or coagulopathy, is the main characteristic of the disease. However, atypical distributions of purpura over the face, neck, and upper extremities have also been observed.^4^, ^14^ In such cases, other differential diagnoses such as immune thrombocytopenia purpura, coagulopathy, drug reactions, leukemia, or other types of vasculitis should be considered.^15^ Affected patients may have three other significant presentations: arthralgia or arthritis, GI involvement with abdominal pain or bleeding, and renal disease.^16^ Kidney involvement is the most crucial factor affecting disease outcomes, with disease severity varying between children and adults. The severity of kidney disease may range from transient microhematuria and non‐nephrotic proteinuria to nephritic or nephrotic disease. In children, IgAVN develops in a median of 30% of cases,^2^, ^3^, ^4^ with the potential for remission in most instances, but a risk of progression in 5%–15% of cases.^2^, ^4^ In global population studies, adults diagnosed with IgAVN demonstrated markedly more pronounced renal dysfunction than that observed in children.^17^ Renal involvement is prevalent in adults, occurring in a median range of 50%–80% of initial cases. This is associated with a notable risk of kidney failure, hypertension, vasculopathy, and cardiovascular mortality.^18^, ^19^, ^20^ No specific risk factors or biomarkers have been identified for the development of IgAVN.

**FIGURE 1:**
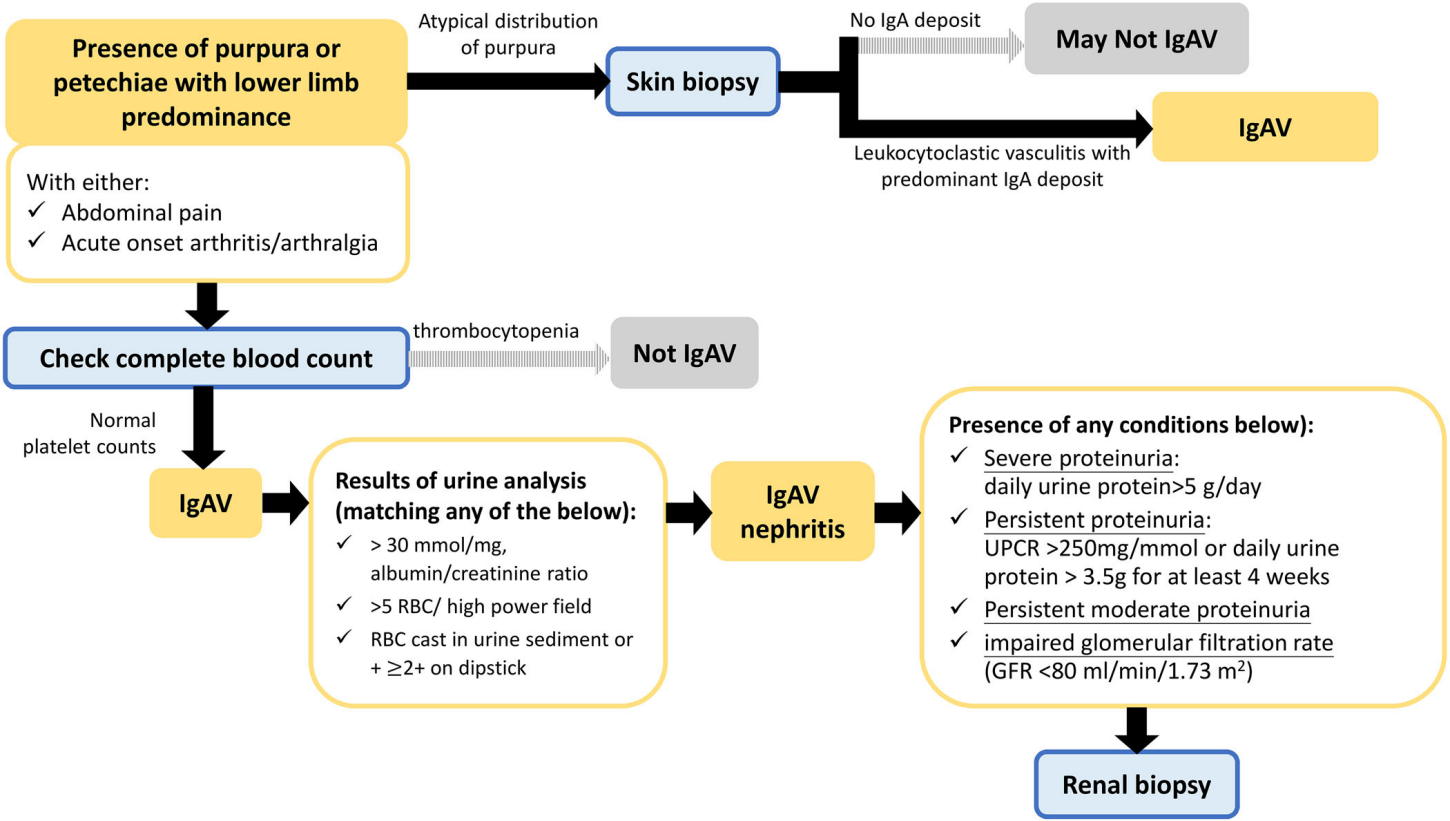
Flowchart of diagnostic strategy for IgA vasculitis. The flowchart is modified by the SHARE‐initiated European consensus‐based recommendations.^24^ IgAV, IgA vasculitis; RBC, red blood cell; UPCR, urine protein‐to‐urine creatinine ratio.

The diagnosis of IgAV was initially guided by the 1990 American College of Rheumatology (ACR) criteria or the 2010 Pediatric Rheumatology European Society classification criteria,^21^ which demonstrated a sensitivity of 87.1% and specificity of 87.7% in patients under 20 years of age.^22^ Later, new criteria were jointly developed by the European League Against Rheumatism (EULAR), Pediatric Rheumatology International Trials Organization (PRINTO), and Pediatric Rheumatology European Society (PRES) in 2010,^23^ which included more clinical features of IgAV. These criteria offer better sensitivity and specificity for diagnosing IgAV in both children and adults compared to the ACR criteria.^22^ The EULAR/PRINTO/PRES‐endorsed criteria are currently included in the European consensus‐based recommendations for diagnosing IgAV.^24^ To rule out other potential diagnoses in atypical cases, physicians should consider skin biopsies with targeted immunofluorescence for IgA. Although kidney biopsy is the gold standard for diagnosing IgAVN, it is particularly recommended if a patient presents with severe proteinuria (urine protein creatinine ratio, UPCR >250 mg/mmol, equivalent proteinuria >3.5 g daily for at least 4 weeks), persistent moderate (UPCR 100–250 mg/mmol) proteinuria or impaired glomerular filtration rate (GFR < 80 mL/min/1.73 m^2^).^24^ An epidemiological survey of children with IgAVN in Japan recommended performing a relatively early renal biopsy when the patients demonstrated kidney dysfunction^25^ and a two‐fold increase in serum creatinine levels, regardless of the severity of proteinuria. A biopsy should be considered in patients without kidney dysfunction but with significant proteinuria. The diagnostic strategies for IgAV and IgAVN are displayed in Figure 1.

### 2.3 Disease course and outcomes of IgAV

Generally, patients with IgAV, especially pediatric patients, have a benign disease course. Most patients have a self‐limiting disease or attain remission within 4 weeks.^1^ Based on various definitions of recurrent disease, recurrent IgAV has been reported in 2.7%–32.6% of pediatric groups^3^, ^26^, ^27^, ^28^, ^29^ and 11%–43.3% of adult groups.^17^, ^28^, ^30^, ^31^ Our preliminary analysis of the NHIRD in Taiwan demonstrated that 5.2% of pediatric patients with IgAV experienced a treatment‐requiring recurrent episode. The most common presentation at relapse was a cutaneous manifestation. Previous studies have revealed that the risk factors for recurrent or relapsing IgAV include older age at onset in children,^3^ younger age in adults,^17^ prolonged duration of illness,^26^, ^28^ lack of corticosteroids at diagnosis,^17^ IgAVN,^3^, ^26^, ^27^, ^32^, ^33^ and joint involvement.^26^, ^28^ The most common predisposing factor for relapse is upper respiratory tract infection in both children and adults with IgAV.^31^, ^34^ In previous reports, most patients, particularly pediatric patients, had mild disease at relapse. A large cohort study reported that 82.7% of patients with relapsing IgAV fully recovered, while 8.3% experienced renal sequelae including persistent nephritis.^28^ However, another study targeting 67 adult patients with IgAV reported progressive disease patterns in 21% of relapsing cases over a 4‐year follow‐up period. This suggests that physicians should pay attention to recurrent disease in adult patients and provide timely management.

Although uncommon, refractory or persistent IgAV is another challenge for physicians treating this disease. Patients with persistent disease may present refractory arthritis, GI symptoms, or renal presentations. In pediatric IgAV, 11%–15.7% of patients present with persistent disease.^3^, ^35^ In contrast, adult patients present with a high persistent course ratio, ranging from 21% to 39%^17^, ^31^, ^35^ and often experience a severe disease. Among the features of IgAV, ongoing renal disease is the most common persistent organ manifestation and is critical for long‐term prognosis.^2^, ^3^ Several factors have been suggested for possible refractory disease in the pediatric group, including renal presentations and older age at onset, which may be confounding factors for both renal and persistent disease development.^3^, ^36^ Persistent renal involvement can result in long‐term kidney damage. Hoˇcevar et al. reported the disease outcome in 362 adults with IgAV after a 127‐month observation. The 74 (27.9%) patients with persistently abnormal urinalysis had an increased incidence rate ratio of 2.27 (95% confidence interval [CI] 1.16–4.40; *p* = 0.012) compared to the ratio in those without apparent IgAVN. The study also examined other risk factors for eGFR decline, including older age, preexisting eGFR <60 mL/min, and persistent abnormal urinalysis, all of which were identified as significant.^17^ Some other predictors demonstrated a correlation to long‐term chronic kidney disease or end‐stage renal disease, including severely impaired renal function, heavy proteinuria, nephrotic syndrome at onset, or nephrotic syndrome with a duration of over 3 months.^2^, ^18^ Monitoring urine analysis within 6 months of IgAV diagnosis and providing adequate treatment are essential in managing cases with IgAV, especially in adults and those who develop renal insufficiency.

## 3 UPDATE OF PATHOPHYSIOLOGY IN IgAV

### 3.1 Trigger factors of IgAV

Although the cause of IgAV remains unclear, previous studies have implicated infectious triggers. Several epidemiological studies have demonstrated seasonal tendencies in pediatric IgAV, which vary across different countries^3^, ^9^, ^37^, ^38^ and are linked to certain pathogen epidemics.^37^, ^38^ Several studies have explored the correlation between specific pathogens and IgAV (Table 1). Several hypotheses exist for the pathogens that induce IgAV; however, these remain inconclusive. The ‘molecular mimicry’ theory posits that certain microorganisms may possess epitopes similar to those in the human small blood vessel wall, further leading to vessel inflammation and damage.^39^ Another group of microorganisms that may play a role in the pathogenesis of IgAV is the commensal microbiome of the human body, particularly in the mucosa. An increasing number of studies have focused on the relationship between the human microbiota and autoimmune diseases.^40^ To date, no conclusions have been reached regarding the correlation between IgAV and microbiota. A few studies have reported that dysbiosis in the gut, but not in the oral cavity, is associated with IgAV. Moreover, the serum IgA levels are correlated with the abundance of some flora.^41^, ^42^ However, the evidence for the role of the microbiome in IgAV remains weak, and further exploration of the mucosal microbiota is required.

**TABLE 1:** Pathogens associated with IgA vasculitis.

		References
Bacterial infections	*Streptococcus pyogenes*	37, 43, 44
	*Streptococcus pneumoniae*	37
	*Staphylococcus aureus*	45
	*Helicobacter pylori*	46, 47
	*Haemophilus parainfluenzae*	48, 49
	*Mycoplasma pneumoniae*	50
Viral infections	Parainfluenza virus	51
	Influenza	38
	Human rhino enterovirus	37
	Rotavirus	38
	Cytomegalovirus (CMV)^a^	52, 53
	Epstein–Barr virus (EBV)	54
	Hepatitis A, B, and C viruses	55
	SARS‐CoV‐2	56, 57, 58, 59, 60
Parasite and yeasts	Cryptosporidium	61
	Giardia	61
Vaccination	Hepatitis A and B viruses	62
	Influenza	63, 64
	SARS‐CoV‐2	65, 66, 67

^a^ CMV is not the trigger of IgAV but is associated with disease severity.

### 3.2 Role of IgA in IgAV pathogenesis

**FIGURE 2:**
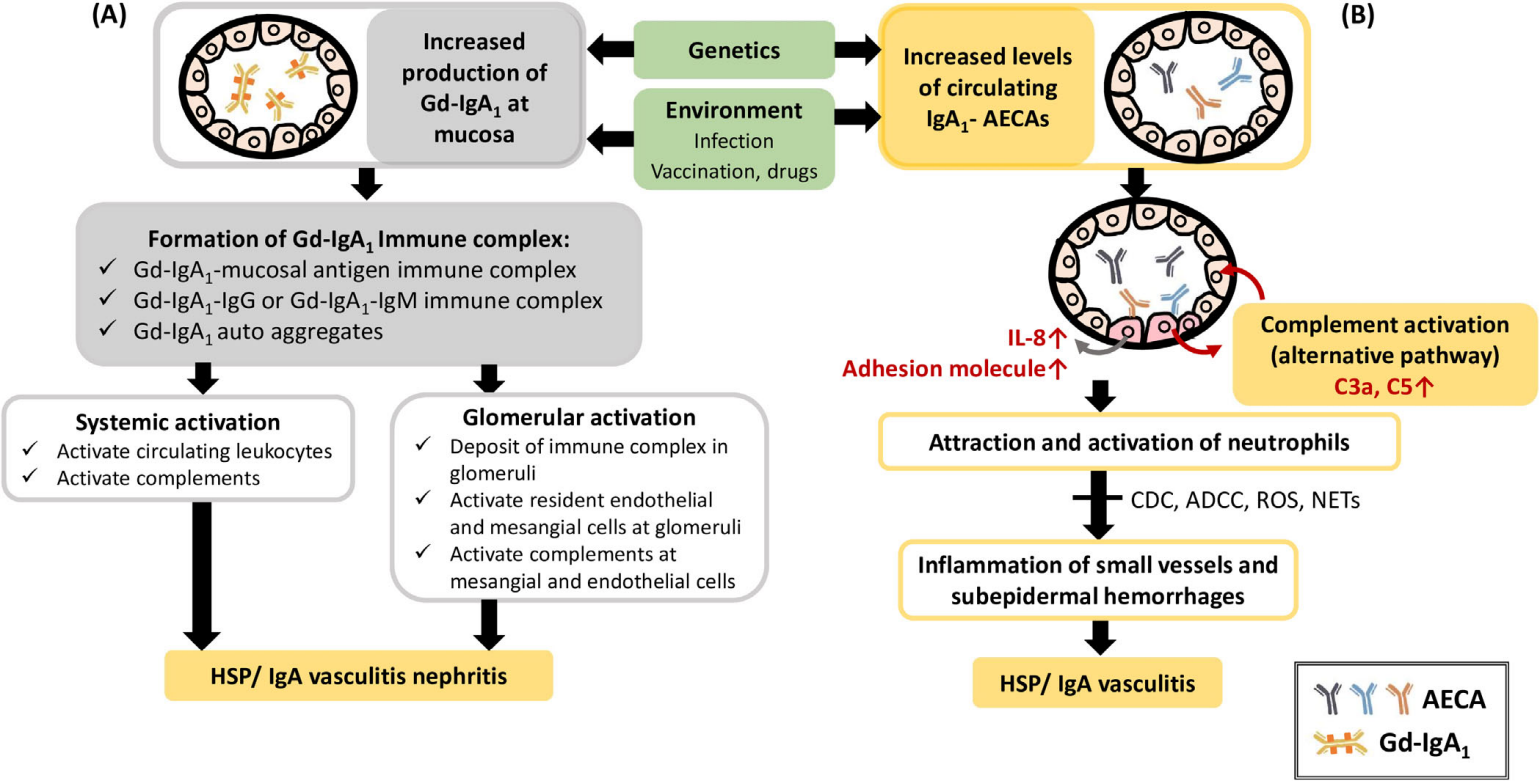
Multi‐hit pathogenesis model for IgA vasculitis and IgA vasculitis nephritis. (A) The mechanism of aberrantly glycosylated IgA; (B) the mechanism of anti‐endothelial cell antibody formation. ADCC, antibody‐dependent cellular cytotoxicity; AECA, anti‐endothelial cell antibody; CDC, complement‐dependent cytotoxicity; Gd‐IgA1, galactose‐deficient IgA1; IL, interleukin; NETs, neutrophil extracellular traps; ROS, reactive oxygen species.

IgA plays a critical role in the immunopathogenesis of IgAV, as evidenced by increased serum IgA concentrations in half of the patients, IgA‐containing circulating immune complexes (CICs), and IgA deposition in vessel walls and renal mesangium in previous case studies.^68^, ^69^ Furthermore, IgA is a primary class of antibodies present in mucosal secretions and serves as the first line of defense against pathogenic invasion. Little is known about the pathophysiology of IgA in autoimmune diseases. However, IgAVN is believed to be pathologically related to IgA nephropathy (IgAN) due to their similar renal histopathology patterns, elevated systemic IgA levels, and CICs. Over the past 20 years, elevated serum levels of aberrantly glycosylated, specifically galactose‐deficient IgA1 (Gd‐IgA1) in O‐linked glycans of its hinge region, have been observed in IgAN.^5^, ^6^ More than 70% of patients with IgAN demonstrated increased serum Gd‐IgA1 levels compared to those in healthy controls.^70^ Similar findings have been observed in patients with IgAVN.^71^, ^72^, ^73^ In patients with IgAN, B cells have been reported to demonstrate decreased levels of β1,3‐galactosyltransferase, an enzyme that attaches galactose to N‐acetylgalactosamine (GalNAc) as well as reduced levels of a molecular chaperon, necessary for galactosyltransferase stabilization. This affects the formation of Gd‐IgA1.^74^ Antibodies against GalNAc‐containing molecules expressed on bacterial or viral structures may cross‐react with aberrant IgA, such as Epstein–Barr virus, respiratory syncytial virus, herpes simplex virus, and streptococci,^75^, ^76^ although direct evidence for this remains lacking. These mechanisms (Figure 2A) might elucidate the pathogenesis in some patients with IgAV. However, the levels of Gd‐IgA1 were not statistically correlated with disease severity or clinical and pathological features.^73^

Another mechanism of action of IgA is the formation of anti‐endothelial cell antibodies (AECAs) in IgAVs,^77^ as illustrated in Figure 2B. Previous studies have demonstrated that the IgA autoantibodies identified during the acute phase included AECAs.^78^ These antibodies can bind to certain molecular antigens on the surface of endothelial cells, such as cardiolipin^79^ or β2 glycoprotein I (β2GPI).^80^ Infection is thought to trigger the production of cross‐reactive AECAs that target antigens on blood cell walls, sharing molecular structures with certain microorganisms.^81^ In in vitro experiments, AECAs led to vessel damage via antibody‐dependent cell cytotoxicity or complement‐dependent cytotoxicity after combining with antigens on the surface of endothelial cells.^78^ Pathological CICs of AECAs lead to the recruitment and migration of neutrophils via increased levels of interleukin‐8 and leukotriene B4. This process further damages the vascular endothelium. Nevertheless, confirming specific antigens on endothelial cells remains challenging, and consistent antibodies targeting endothelial cell molecules such as β2GPI, are only present in some patients with IgAV.^76^, ^80^ This phenomenon revealed the heterogeneous disease‐triggering factors and complexity of IgAV.

### 3.3 Role of neutrophils in IgAV pathogenesis

The importance of neutrophils and their roles in the pathogenesis of IgAV have been widely explored in recent years. As a leukocytoclastic vasculitis, IgAV has been associated with an increase in absolute neutrophil levels or the neutrophil/lymphocyte ratio (NLR) in patients with IgAV compared to that in healthy controls.^82^, ^83^, ^84^, ^85^, ^86^ In addition, an increase in NLR, as a biomarker of IgAV, may serve as a predictor of GI inflammation in IgAV.^82^, ^84^, ^85^ Chen et al. reported several findings on the association between neutrophil extracellular traps (NETs) and disease activity in IgAVs.^87^ Serum from patients with IgAV at different disease stages revealed an increase in the circulating levels of NETs and a decrease in the degradation of NETs in patients with active IgAV. In addition, the study detected the presence of NETs in the kidney and GI tissues of patients with IgAV using Immunofluorescence staining.

Regarding the associations between IgA and neutrophils in IgAV, Mayer‐Hain et al. elucidated the role of polymorphonuclear neutrophils (PMNs) in mediating vessel damage through NET formation in response to IgA immune complexes (IgA‐ICs).^88^ They discovered significantly elevated serum concentrations of IgA‐ICs comprising IgA/IgG than those in controls. In addition, IgA from the serum of patients with IgAV induces significant DNA release from PMNs. Moreover, during acute flares. IgA binds to the surface of peripheral PMNs in patients with IgAV, a phenomenon not observed in healthy controls.

## 4 UPDATE OF ASSOCIATION BETWEEN IgAV AND COVID‐19

Severe acute respiratory syndrome coronavirus 2 (SARS‐CoV‐2) infection originated in Wuhan, China in late December 2019 and quickly spread worldwide.^89^ On March 11, 2020, the World Health Organization officially classified COVID‐19 as a global pandemic. With the spread of SARS‐CoV‐2, several case reports or series on COVID‐19‐related IgAV have been published. Among all COVID‐19‐related vasculitis, IgAV is one of the most commonly reported systemic vasculitis induced by SARS‐CoV‐2 infection or vaccination.^65^, ^67^ Several systematic reviews on COVID‐19‐related IgAV demonstrated that IgAV triggered by SARS‐CoV‐2 affected adult patients predominantly and tended to have a better prognosis than that of de novo systemic vasculitis, with a favorable response to glucocorticoids with or without immunosuppression.^65^, ^67^ However, the disease course in children with COVID‐19‐related IgAV is not usually benign or self‐limiting.^7^ In an international multicenter study, Batu et al. analyzed the features, treatments, and disease courses of patients with COVID‐19‐associated pediatric vasculitis, excluding multisystem inflammatory syndrome in children, a Kawasaki disease‐like vasculitis related to COVID‐19.^90^ Among the 41 patients with COVID‐19‐related vasculitis, 30 were diagnosed with IgAV. Compared with the characteristics of patients with IgAV from the pre‐pandemic cohort, more children with COVID‐19‐associated IgAV presented with fever (30%) and renal involvement (43.3%). In addition, the report indicated that children with COVID‐19‐associated IgAV may have experienced a more severe disease course than that observed in pediatric patients with IgAV before the pandemic.

The pathogenesis of COVID‐19‐related vasculitis is expected to be complex and potentially involves interactions between viral components and host immune responses. Vasculitis can be caused directly by viral damage, or indirectly by the immune response triggered by SARS‐CoV‐2 infection. Histopathological studies have demonstrated that SARS‐CoV‐2 directly causes endotheliitis, with evidence of viral inclusions in endothelial cells and increased endothelial apoptosis in patients with COVID‐19.^91^ By binding to the angiotensin‐converting enzyme 2 (ACE2) receptor, SARS‐CoV‐2 downregulates ACE2, leading to elevated angiotensin II (Ang II) levels,^92^ and decreased nitric oxide (NO).^14^ Increased Ang II and NO levels contribute to vasoconstriction and vascular inflammation. These changes can trigger a shift in macrophages to a pro‐inflammatory M1 status, resulting in vascular inflammation.^93^, ^94^ In addition, SARS‐CoV‐2 triggers an extremely strong immune response and leads to a cytokine storm,^95^, ^96^ which may lead to a more severe disease course in COVID‐19‐associated IgAVs than in traditional cases. Similar to other IgAV pathogens, the mechanism by which COVID‐19 directly causes IgAV infection remains uncertain. However, some clues exist, such as the sustained presence of neutralizing anti‐SARS‐CoV‐2 IgA antibodies from the early phase of the infection.^97^ Although a lack of evidence exists for increased Gd‐IgA1 in COVID‐19‐related IgAV, an increase in anti‐cardiolipin and anti‐β2GPI IgA has been identified in severe COVID‐19 cases.^98^

Although COVID‐19‐related IgAV has been reported, limited studies from Korea, Japan, and Turkey have noted a decrease in newly diagnosed cases with IgAV during the COVID‐19 pandemic.^58^, ^99^, ^100^ The pandemic has brought about significant changes in our daily routines, including the adoption of non‐pharmaceutical interventions (NPIs) such as wearing masks, social distancing, quarantine, and curfews. These measures have contributed to a reduction in the transmission of other infectious agents. IgAV has been considered infection‐related as preceding upper respiratory infections have been reported in 50%–60% of patients with IgAV.^3^, ^10^ Thus, the primary reason for the decrease in the diagnostic rate of IgAV could be attributed to a decline in the frequency of upper respiratory tract infections other than COVID‐19. A large prospective national surveillance cohort in France involving 9790 children with IgAV and other infectious diseases from 2015 to 2023 revealed a significant decline in the incidence of IgAV after the implementation of NPIs and a remarkable increase after the relaxation of NPIs.^37^ A similar reduction in the incidence of Kawasaki disease has also been observed during the COVID‐19 pandemic,^58^, ^99^, ^100^ suggesting a possible link to infection, similar to other common pediatric vasculitides. The experiences from cases of COVID‐19‐related IgAV as well as the histopathological findings will aid physicians in improved understanding of the pathogenesis of IgAV.

## 5 CONCLUSIONS

IgAV is a multifactorial vascular disease and the epidemiological features of the infection highlight the impact of environmental factors. Most patients demonstrate a favorable prognosis; however, atypical cases may require histopathological examination. For instance, skin biopsy of atypical cutaneous lesions or renal biopsy for persistent or severe IgAVN. Continuous follow‐up and awareness of relapse and refractory disease in patients with IgAV, particularly adults, is an issue for physicians.

However, the pathogenesis of IgAVN is not fully understood. This mechanism is similar to that of IgAN, in which the dysregulated immune response of the body is activated by precipitating events, such as infection, leading to the overproduction of Gd‐IgA1 and Gd‐IgA1‐containing immune complexes. The shared pathways of IgAN and IgAVN suggest a common underlying pathophysiology involving aberrant IgA production, immune complex formation, and renal deposition. Given these shared mechanistic features, experimental targeted treatments developed for IgAN may hold promise for application in IgAVN. Furthermore, another mainstream pathogenic hypothesis involves the formation of AECAs, which can induce endotheliitis and downstream immune response. Neutrophils play a significant role in IgAV pathogenesis. IgA‐ICs promote NET formation, which is associated with IgAV disease activity. These findings highlight the complex interplay between various factors involved in the pathogenesis of IgAV, including pathogens, Gd‐IgA1, AECAs, and neutrophils. Further research is required to fully understand the mechanisms underlying vasculitis development.

## CONFLICT OF INTEREST STATEMENT

All authors declare no conflict of interest.
